# Semi-Supervised Segmentation of Ultrasound Images Based on Patch Representation and Continuous Min Cut

**DOI:** 10.1371/journal.pone.0100972

**Published:** 2014-07-10

**Authors:** Anca Ciurte, Xavier Bresson, Olivier Cuisenaire, Nawal Houhou, Sergiu Nedevschi, Jean-Philippe Thiran, Meritxell Bach Cuadra

**Affiliations:** 1 Department of Computer Science, Technical University of Cluj-Napoca, Cluj-Napoca, Romania; 2 Signal Processing Laboratory (LTS5), École Polytechnique Fédérale de Lausanne, Lausanne, Switzerland; 3 Department of Radiology, University Hospital Center and University of Lausanne, Lausanne, Switzerland; 4 Center for Biomedical Imaging, Signal Processing Core, Lausanne, Switzerland; 5 Swiss Institute of Bioinformatics (SIB), University Hospital Center and University of Lausanne, Lausanne, Switzerland; University of Navarra, Spain

## Abstract

Ultrasound segmentation is a challenging problem due to the inherent speckle and some artifacts like shadows, attenuation and signal dropout. Existing methods need to include strong priors like shape priors or analytical intensity models to succeed in the segmentation. However, such priors tend to limit these methods to a specific target or imaging settings, and they are not always applicable to pathological cases. This work introduces a semi-supervised segmentation framework for ultrasound imaging that alleviates the limitation of fully automatic segmentation, that is, it is applicable to any kind of target and imaging settings. Our methodology uses a *graph of image patches* to represent the ultrasound image and user-assisted initialization with labels, which acts as *soft priors*. The segmentation problem is formulated as a continuous minimum cut problem and solved with an efficient optimization algorithm. We validate our segmentation framework on clinical ultrasound imaging (prostate, fetus, and tumors of the liver and eye). We obtain high similarity agreement with the ground truth provided by medical expert delineations in all applications (94% DICE values in average) and the proposed algorithm performs favorably with the literature.

## Introduction

Ultrasound (US) imaging provides a high image resolution and simplicity of use at much lower cost in comparison with other medical imaging modalities such as Magnetic Resonance imaging (MRI) or Computer Tomography (CT). These advantages make ultrasound a main image modality in medicine and its use becomes of particular interest in areas such echocardiography [1]–[3], obstetrics and gynecology [4], breast cancer [5] and intravascular diseases [6]. Indeed, US imaging is more and more used for image-guided therapy planning and navigation, and computer aided diagnosis. To this end, the development of efficient US image segmentation techniques of biological structures (e.g. different organs, heart chamber, fetus) or focal diseases (e.g. tumors, cysts) is needed.

US segmentation is very challenging due to the inherent speckle and some artifacts like shadows, attenuation and signal dropout. This often leads to weak (or missing) edges and also to the presence of fake edges, making standard unsupervised segmentation methods fail. Indeed, segmentation methods succeed on US imaging only when making use of application-specific constrains or priors. Several types of priors have been suggested in the literature [7]: those coming from *time* or *functional* data and those based on *shape* or *imaging physics*. In this work, we segment 2D B-mode US images without contrast enhancement. In consequence, we will not discuss the use of *time*-based constraints from different imaging frames or *functional*-based priors provided by contrast agent. However, such kind of priors could be included in our formulation if needed.

Anatomical shape priors are powerful for US image segmentation dealing very well with shadows and weak edges and they have been successfully used for different anatomical structures segmentation such as the heart [2], [3], the prostate [8], [9], the breast [10] or the kidney [11]. These priors can be encoded in the form of statistical shape models, usually derived from large data set segmentations, and they are often computationally expensive. Unfortunately, they are of limited use for pathological cases due to high variation in structure, shape, size and localization of lesions. Shape priors can be encoded in a simpler manner as for instance imposing a smooth boundary [2]. We will have such regularization in our approach.

Priors can also be related to the imaging physics, i.e. related to the observed intensities of the ultrasound image. For example, we consider intensity-based priors or learned texture as specific parametric intensity models to characterize the observed data. In this context, two different philosophical branches can be identified: those that consider speckle as information and those that consider it as noise. Since the gray level intensities in US images reflect the tissue density, some approaches use denoising filters in order to reduce the speckle structure and smooth the images (e.g. [12]). On the contrary, other approaches benefit of the texture information contained by speckle. As proved by Oosterveld et al. [13] by realistic simulations, the statistical and speckle characteristics of echographic texture change according to the density and spatial distribution of scatterers within the resolution cell.

A wide range of image features are used for modeling US observed data (see [7] for an extended review). Intensity features are often used by means of analytical models of the gray-level distribution (Rayleigh distribution being the most used model [4] but also Exponential, Gamma and Gaussian). Note that all these models are well-suited for the received signal while, in practice, clinical ultrasound devices log-compress the signal before visualization [14]. The main drawbacks of these gray-level distribution models are related to distribution parameter estimation and to its dependency on the imaging system settings such as dynamic range and gain. Intensity gradient features can also be used [1]. However, they strongly assume that there are homogeneous regions and thus, a very low level of speckle noise. Texture features have also proved to be successful in US segmentation, particularly statistical patterns of the intensity due to their advantage of being independent of the imaging system physics [5].

### Contribution of this work

We consider in this work an alternative approach for characterizing the ultrasound data. Rather than using analytical distribution models or texture patterns to represent ultrasound images, we make use of *graph of intensity patches* as image representation. In contrast with most intensity or texture-based methods, the use of patches as image features allows us to be more independent of the imaging system and no assumption on the echogenicity of the object has to be done. Our choice of patch feature is also supported by previous works in the literature. Patch features were first introduced for texture synthesis [15] and image denoising [16] for natural images. In [17], Coupé et al. extended the non-local means filter [16] to reduce speckle noise in US images, by defining a particular similarity measure between patches. In [18], [19], the patch-based approach was also successfully used for spatio-temporal registration and for motion or/and elasticity estimation in US image sequences of the heart. As far as we know, our preliminary work [20], published in 2011, was the first paper that introduces the use of intensity patches in US segmentation. Besides, we use the Pearson distance between patches as it proves to be a robust distance to speckle, as shown by [17].

Given the graph US image representation, we then address the segmentation problem with an efficient and *interactive* extraction algorithm of the foreground object, where the background cannot be trivially subtracted. Our segmentation method is thus fundamentally semi-supervised, that is, initial *labels* are defined on the image, acting as *soft priors*. Interactive soft priors were introduced in Computer Vision with user-assisted segmentation algorithms such as *Interactive Graph Cuts* [21], Lazy Snapping [22] and *GrabCut* [23]) and also in Medical Imaging (like CT [24] and US [12] segmentation. This quick and easy way to interact with images can provide the priors needed to make our segmentation accurate, robust and applicable to different kind of targets or imaging parameters. From the interaction point of view, our algorithm may be equivalent to a large variety of US segmentation methods proposed in the literature. Several state-of-the-art methods [7] require initial clicks or other types of interaction, like defining a region of interest or much tedious manual training. Moreover, depending on the application's specificities, the label initialization can be automatized, as in [20] for retinoblastoma segmentation.

Our segmentation method is based on the patch-based continuous graph cut approach for natural image segmentation introduced in [25]. Continuous graph cut methods have seen a rapid development over the recent years [26]–[32]. These methods find their theoretical roots in Strang [33] but the interest of applying them to real-world applications like medical imaging and computer vision has been triggered only recently. Nevertheless, continuous graph cut methods are quite attractive and offer new features that we will discuss in a further section.

A preliminary version of this work was presented in [20]. The main differences and improvements of the proposed work with respect to our previous work are as follows. Firstly, we introduce a new minimization algorithm that speeds up by at least an order of magnitude our previous optimization algorithm proposed in [20]. Secondly, we perform several tests of the proposed ultrasound segmentation methodology to identify clinical targets, namely, prostate, fetus, liver tumors and eye. Eventually, we carry out a thorough study of the robustness of our segmentation algorithm with respect to initialization.

As shown in [7], most US segmentation methods are usually limited to a specific target (as for instance the endocardial border [2], [3], [34], [35], breast mass [5], [36]–[38], prostate [8], [12], [39], [40], and liver [41], [42]). The major advantage of our segmentation framework, as regards the state-of-the-art, is its flexibility and easiness to use, while obtaining equivalent or better accuracy. However, this prevents us from selecting one approach in the literature for comparison that would not be equivalent in terms of flexibility. We will thus focus on one clinical context for method comparison, the segmentation of the prostate. This is one of the main areas of application of US in cancer treatment [40]. We have chosen the most recent, and methodologically close, semi-supervised approach presented in the literature [12], that makes use also of graph theory and soft priors using initial labels.

We now summarize the main methodological contributions of the proposed US segmentation method:

- An efficient representation of US images based on graph of intensity patches (naturally adapted to any echogenicity and imaging systems),
- A fast minimization algorithm for the minimum cut problem,
- An easy use of soft priors based on interactive user labels (unlike hard priors such as shape or temporal constrains),
- A study of accuracy and reproducibility of our algorithm with respect to different initial labels and users;

and the main application-oriented contributions are:

- A flexible framework, applicable to different US segmentation problems (eye, liver, prostate and fetus),
- A high accuracy of segmentation results as compared to manual delineations of expert raters, with mean Dice values around 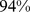 in all data sets.

A related work on ultrasound image segmentation were recently introduced in [43], [44] in the context of echocardiographic sequences. This work also promotes the use of intensity patches for ultrasound segmentation. More precisely, the authors of [43], [44] define a sophisticated image representation model with multiscale signal analysis and sparse coding technology, which make the image representation rich but also time-consuming. We directly use image patches as image representation and show that this simple image representation is quite flexible and accurate to deal with multiple segmentation scenarios. Besides, [43], [44] make use of the level set method (LSM) as segmentation method. The LSM is a PDE-based segmentation technique, which speed is limited by the Courant-Friedrichs-Lewy (CFL)'s condition. Discrete and continuous graph cut techniques (like the one introduced in this work) are more recent segmentation techniques and are not limited by the CFL condition, thus faster than the LSM (at least by an order of magnitude).

The proposed paper is organized as follows. In the first section, our mathematical framework and minimization scheme are presented. In the second section, the parameter setting and datasets are introduced. In the third section, we study the accuracy, repeatability and computational speed with respect to the initialization step. Then, quantitative results are presented for liver, eye tumors, fetal head estimation, and prostate. Eventually, a discussion is presented in the last section.

## Methodology

**Figure 1:**
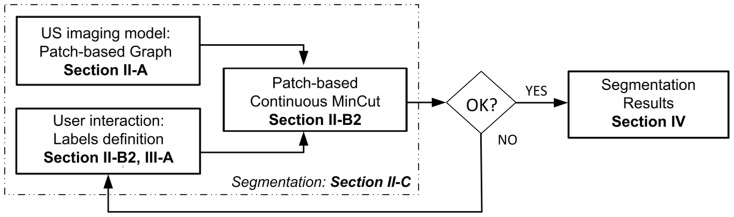
Our segmentation framework.

The general block diagram of the proposed US segmentation method is shown in Figure 1. With a given US image as an input, the method first models the image with a graph representation of patches; next, the user provides markers inside (and outside, if needed) the object as initialization, serving as method initialization; finally, the novel numerical scheme for the minimization process is applied. Note that, in practice, if the segmentation result is not satisfying, our framework offer the possibility to re-initialize the labels and segment again, without re-computing the image modeling step. We will denote our new fast Patch-based Continuous Min-Cut segmentation by *fP-CMC* to distinguish it from our previous version *P-CMC* in [20].

### Modeling of US images

Let an image 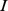 be defined as a vector in 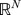 where 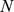 is the number of pixels in the image. In this work, we represent US images as a graph of image patches. Therefore we introduce a graph 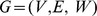, where the set 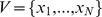 contains the nodes of the graph, the set 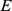 contains the edges (i.e. the connections between the nodes) and 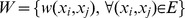 is the weight matrix that encodes the similarity between two points (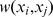 is small when 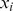 is different from 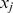 and 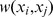 is large when 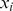 is similar to 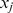). Each node 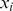 of the graph 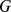 represents a pixel in the image 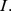 The following graph weight is considered:
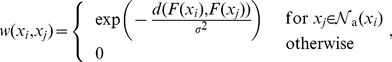
(1)where 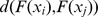 expresses the proximity between the image descriptors located at 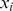 and 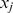, 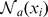 is a neighborhood window centered at 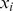 of size 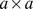, and the parameter 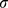 is the scaling parameter of the weight matrix (using a standard Gaussian kernel with 0-mean and variance 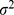). In our case, the image descriptors 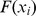 and 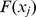 are 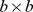 image patches centered at 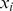 and 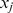, respectively. Note that the proposed segmentation framework is flexible enough to support any types of image descriptor. A standard distance 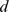 that measures the similarity between image patches is the 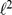 norm, a.k.a. the mean square difference, as used in [25] for natural image segmentation. Our patch feature is more adapted for US image segmentation than most texture descriptors used in the literature in several ways: it does not need multiple resolution characterization, it does not need any adjustment for ultrasound imaging (except from the patch size that can be easily set), it has no trouble in segmenting small areas (as some texture-based methods do) and, it is extremely easy to be computed.

### Speckle model and Pearson distance

In the context of US imaging, patch distance is hard and critical to define. We need to consider the complex image formation of the US images such as local correlation due to periodic arrangements of scatterers and finite beam width, envelop detection and logarithm amplification of radio-frequency signals, and additive Gaussian noise of sensors. Consequently, we choose the following speckle model, that was proved in [45] to fit well the log-compressed US images:
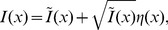
(2)where 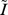 is the original image, 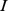 is the observed image and 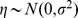 is a Gaussian noise. Thus, for each pixel 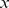 can be made the assumption that
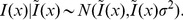
(3)


An extensive study was performed by Coupé et al. [17] that proved the higher performance of the Pearson distance against the L2 norm to measure the similarities between patches in US images. Thus, we choose to use the Pearson distance between patches for segmentation since it is better adapted to the speckle:
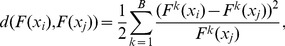
(4)where 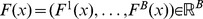, where 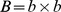 is the number of pixels in the patch 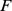 centered at 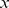 and 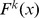 is the intensity of 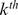 element of the patch.

### Segmentation model

#### Continuous graph cut model

An efficient approach to solve the US segmentation problem is to cast the problem as a *continuous* graph partitioning problem such as [25], [33]. In this work, we design a continuous graph cut model to carry out the US segmentation task with soft label priors.

Continuous graph cut methods, such as see [26]–[32], have seen a rapid development in the recent years based on new attractive properties. Different from discrete graph cut methods such as [21], [23], [46], [47], which are generaly based on combinatorial optimization techniques, continuous graph cut techniques are fundamentally different: they are built on continuous tools like elliptic Partial Differential Equation (PDEs), variational methods and continuous convex optimization techniques like augmented Lagrangian, Uzawa-type primal-dual, iterative shrinkage techniques, etc. Mathematical fields like Functional Analysis offer strong tools to analyze the well-posedness of these continuous graph cut techniques. The main advantages of the continuous formulation of the graph cut methods are 1) sub-pixel accuracy, 2) easy to code (a few lines of Matlab) and 3) easy to parallelize with significant speedups.

Let us now introduce our continuous graph cut methodology, which benefits from these mentioned new features. Graph partition methods aim at segmenting a graph 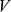 into two subsets, 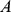 and 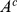, such that 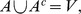 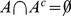 and the inter-similarity between 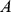 and 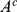 being minimized. Partitioning a graph into two sets 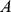 and 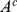 can be achieved by minimizing the cut operator defined as:
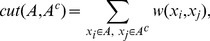
(5)which is equivalent to:
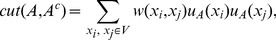
(6)
*when* 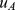 *is an indicator function of the set A*. More specifically, we have 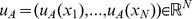 with 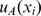 is the 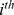 element of the vector 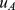 and 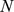 is the number of pixels in the image. Function 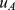 is an indicator function of the set 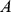 when 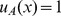 for 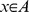 and 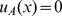 for 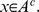 It is known that minimizing the cut operator (5) is equivalent to minimizing the graph-based 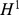 norm as long as binary indicators, i.e. 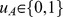, are considered [48]. The graph-based 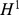 norm is defined as:
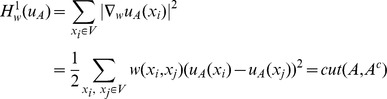


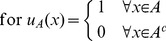
(7)


Observe that the weighted graph Laplacian naturally corresponds to a finite difference approximation of the continuous Laplacian operator and it also corresponds to the non-local operator of diffusion on graph.

#### Segmentation with labels

The proposed segmentation algorithm aims at minimizing the cut operator (or graph-based 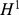 norm) given some labels to identify the objects of interest and the background. The object labels 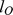 are defined as 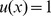 for 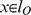 and the background labels 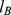 are defined as 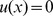 for 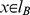. We now formulate the segmentation method as a discrete minimization problem:
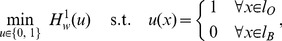
(8)where 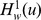 is defined in (7) and 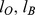 are provided by the user. As Shi and Malik observed in [48], minimizing the cut can favor small sets. An easy way to overcome this issue, while smoothing the irregularities along the cut boundary, is to use the total variation (TV) norm (i.e. the 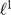 norm of the gradient) as follows:
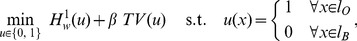
(9)with 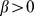, 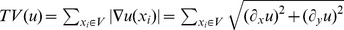 where 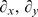 are the discrete spatial derivative operators in the *x,y*-directions. The discrete minimization problem (9) is a combinatorial problem difficult to solve because the set of minimization is the binary function (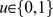) which is not convex. The natural approach is to relax the binary constraint to the closest convex set which is naturally 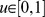. We thus consider the following continuous minimization problem:
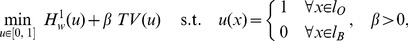
(10)which is convex, therefore providing a unique solution for any initial condition. Another nice consequence of (10) is the opportunity to develop efficient continuous minimization schemes, based on recent development for 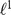 optimization (for the compressed sensing field). The proposed segmentation model (9) was already introduced in [25] (based on [24]). However, we will present in the next section a novel and (much) faster minimization algorithm to solve (10).

### Efficient minimization algorithm

This section introduces a fast minimization algorithm for (10) based on augmented Lagrangian method and splitting technique such as [49]–[53]. The minimization problem (10) is equivalent to (splitting step):
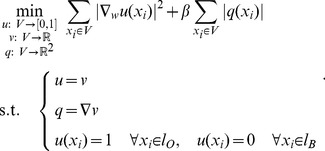
(11)


Adding new variables by a splitting step is a standard approach that can solve a difficult minimization problem by equivalently solving easier sub-minimization problems. Indeed, the constrained minimization problem (11) can be solved by the following iterative unconstrained minimization problems (augmented Lagrangian step):
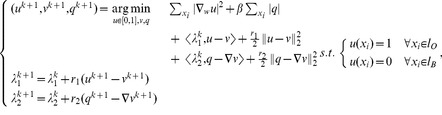
(12)


where 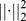 and 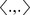 are the standard 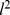 norm and scalar product and 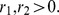 Then, we consider the solution of the three sub-minimization problems in (12). The first sub-minimization problem is:
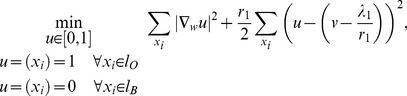
(13)


The Euler-Lagrange equation of (13) is:
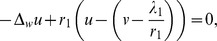


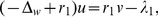
(14)where 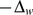 is the graph Laplacian. The linear system of equations (14) can be solved efficiently with a conjugate gradient method (with e.g. Matlab). Then, the constraints 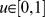 and 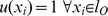, 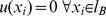 are simply imposed to the solution of (14). The second sub-minimization problem to solve is:
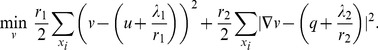
(15)


The Euler-Lagrange equation of (15) is:
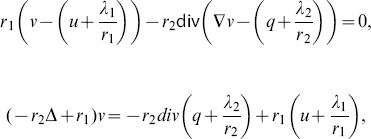
(16)which can be solved quickly with fast Fourier transform (FFT) or discrete cosine transform (DCT) depending on the boundary condition. The third and last sub-minimization problem is:
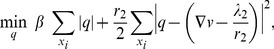
(17)which solution is given by soft-thresholding [54]:
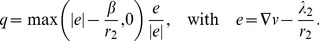
(18)


The pseudo-code is given in Table 1. to summarize the proposed algorithm. Finally, the stopping condition is chosen to be 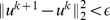 and 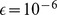 in all experiments.

**Table 1:** Algorithm 1.

**Initialization: ** 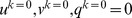
**While** 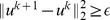 ) **do**
**Compute:** 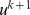 with Eq. (14)
**Constrain:** 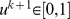 and 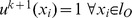 , 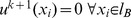
**Compute:** 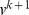 with Eq. (16)
**Compute:** 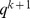 with Eq. (18)
**Update:**
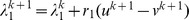
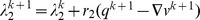
**end while**

Fast optimization scheme for US image segmentation

### Parameter setting

A detailed study of the parameters 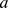, 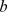 and 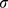 has previously been done in the context of image denoising for natural [55] and ultrasound images [17]. We briefly re-visit here after the parameter setting in the context of B-mode US segmentation.

*Searching window size (* 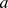 *)* - It has a great impact on the computational time. The size of searching window is also related to the “non-local character” of the method, since the similar patches are chosen inside this window and not only from the nearest neighbors.

*Patch size (* 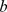 *)* - The patch size is known to be related to the texture pattern size and to the target object scale [17], [55]. In ultrasound, texture pattern is provided by speckle. High values of 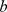 imply an increased computational time of weighted matrix 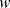 and the segmentation becomes coarser and a loss of details is noticed. Thus, we set 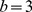 to ensure the highest precision and a fast computation [56].

*Scaling parameter (* 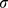 *)* - It stands for the typical distance between similar patches, which depends on the noise level [55]. For the proposed speckle model, according to Eq. (3), we have a level of noise equal to 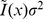. Thus, the selection of 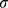 parameter depends on the distribution of the gray levels inside the reference patch (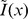). That implies that 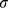 also depends on speckle characteristics such as speckle size and if it is fully developed or not. Based on the same assumption defined in Eq. (3), the level of noise 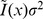 has a high variation because of the speckle appearance. This leads to a low power of discrimination between two different tissues with close density properties (meaning a low contrast in US image). A value of 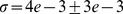 performs goods results in case of US images, with a higher sensitivity for the mentioned cases of tissues with close density properties.

*Regularization weight (* 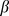 *)* - It controls the smoothness of the contour and eliminates the misclassification of small subsets of pixels resulted by the min-cut algorithm. Optimal results in ultrasound segmentation are performed for 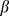 values between 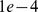 and 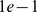. Larger 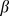 value, smoother the contour is. The sub-parameters 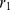 and 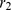 occurred in the augmented Lagrangian step (Eq. 12) are both fixed to 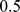.

For all the tests and clinical applications presented in this paper we used the following setting: 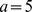, 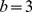, 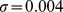 and 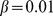.

### Data sets

Our data set contain a wide variety of ultrasound images in order to proof its flexibility and performance for different imaging systems, image resolution, speckle size and targets. It contains a total number of 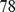 images from which 20 ophthalmic, 30 liver, 22 fetal and 6 prostate images. Table 2 summarize the characteristics of each data set. Besides the US images, we dispose of manual delineation performed by medical experts for the ophthalmic, liver and prostate data sets and of manual biometric measurements for the fetal data set, which were used as ground truth in our tests.

**Table 2:** Data sets overview.

Target		US system	Frequency (MHz)	Estimated speckle size	Isotropic resolution ( 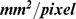 )	No images	Image size
Liver	-depth: 8 cm -depth: 16 cm	Logiq 7	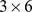 5.5	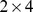	0.2×0.20.4×0.4	1515	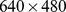
Fetus		Siemens Medical Systems	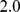 – 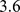	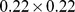 -	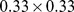	22-	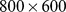
Eye		OTI Ophthalmic Technologies Inc.	12.5	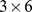	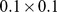	20	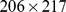
Prostate		-	-	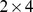	-	6	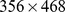

### Ethics Statement

The patient information from all data used in our study was anonymized and de-identied by physicians prior to our analysis. All studies presented are approved by the corresponding committee/institutional review board: Switzerland (Cantonal Research Ethics Committee of Vaud), France (Comite de Protection des Personnes), Romania (local Ethical Committee of the University of Medicine and Pharmacy Cluj-Napoca) and Canada (Health Science Research Ethics Board at Western University).

### Opthalmic imaging

Ultrasound is still in its early years as regards ophthalmology. However, its use is constantly increasing [57], [58] for diagnosis, planning, therapy and follow up of treatments. We present the segmentation of B-scan US of the retinoblastoma. The clinical value of this work is related to a larger project that aims at improving the radiotherapy planning and treatment of retinoblastoma in childhood by fusion of CT, ultrasound and fundus of photograph [59]. Our clinical eye data set contains 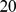 ophthalmic US images which are 2D slices of the 3D volumes from 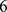 eyes with retinoblastoma (including both calcified and non calcified tumors). US eye images were acquired at *Jules Gonin Hospital*, Lausanne, Switzerland with Ophthalmic Technologies Inc. (OTI), having an isotropic resolution of 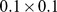 mm^2^ and image size of 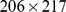 pixels.

### Liver

Liver ultrasound aims at finding abnormalities, such as scarring (cirrhosis), masses (both cancer and non cancer) and blockage of the blood vessels. These findings help determine the diagnosis and therapy and also whether the patient is good or not as transplant candidate. In clinical practice, liver diagnosis requires additional examination with other invasive methods like biopsies, with the associated morbidity and mortality risk. Therefore, the automated segmentation of ultrasound images would provide a reliable non-invasive and quantitative approach in diagnosing liver diseases.

We will present the lesion segmentation of US liver imaging from 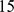 different patients diagnosed with hepatocarcinoma (HCC). These application does not aim at illustrating any clinical value but a quantitative validation thanks to the available manual delineations. The US liver images were acquired at 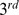 *Medical Clinic*, Cluj-Napoca, Romania using Logiq7 system at a frequency of 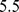
MHz, with isotropic resolution of 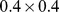 mm^2^ (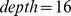 cm) and 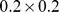 mm^2^ (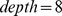 cm) respectively and image size of 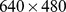 pixels. The data set contains two images per patient, one for each resolution, summing up a number of 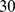 images.

### Fetal imaging

Ultrasound imaging is the gold standard modality for exploration, biometric measurements and diagnosis in fetal imaging. In this context, the development of efficient US image segmentation techniques of biological structures such as the head, femur or abdomen of the fetus are crucial towards a high quality perinatal follow up and diagnosis. Here we aim at segmenting the head of the fetus to estimate later on the head circumference (HC) as an indirect measure of fetal growth. Our data set contains 22 US images from 22 different fetuses ranging from 24 to 35 weeks of gestational age (GA). Images were acquired in clinical practice at *Hôpital Femme Mère Enfant*, Lyon, France with Siemens Medical Systems at 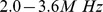 with spatial resolution of isotropic pixels between 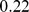 to 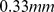. Let us note that only four fetuses can be considered as healthy while the rest are suspected of developmental brain delay. Inner head circumference computed by expert radiologist will be used as gold standard. Our previous method *P-CMC* [20] was evaluated on a different dataset to estimate the outer HC in [60].

### Prostate

Transrectal ultrasound (TRUS) is a key tool for prostate cancer diagnosis. Prostate volumes and boundaries are essential biomarkers in the diagnosis, treatment, and follow up of prostate cancer [7]. This has encourage many researches to develop segmentation tools for prostate boundary detection [40]. The testing data set contains 6 prostate US images, the ones introduced and used for tests in [12].

Let us note that the software and data set for testing presented in this paper will be made publicly available at http://www.unil.ch/mial/page86599.html website upon acceptance of the manuscript.

## Results

### Method evaluation

The overlap measures that we use to estimate the agreement between the segmentation result and the ground truth (GT) are: Dice coefficient metric (DCM) [61] and area overlap (AO, or accuracy overlap) [12]. They are defined as follows:
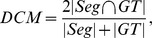
where by 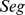 and 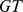 we refer to the set of segmented, respectively ground truth points, and
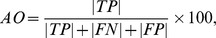



By 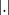 we denote the set cardinal and TP, FN and FP are the *true positive*, *false negative* and *false positive* sets respectively. Added to overlap measures for segmentation agreement, speed in number of iterations and computational time are also shown. Our algorithm is implemented in Matlab and it was tested on an INTEL Core 2 Duo, 2.66 GHz, 2 GB of RAM.

### Robustness to initialization

Our segmentation method is designed in an interactive framework which allows the user to easily and quickly initialize the algorithm for any target of interest. Let us remind that, depending on the specific application, the initialization process can be easily automatized [20]. However, we promote here some initial user interaction in favor of a great gain in flexibility: we will be able to segment many different targets. Having some initial interaction is not new in US segmentation. A large range of state-of-the-art methods must define initial clicks and even heavier interactions (please refer to Table II and III in [7] as regards interaction type for breast and prostate segmentation respectively). In this sense, our algorithm is not different from algorithms in the literature.

To this end, we present a robustness study with different type of initializations: simple straight lines, one click-drag and drop for generating predefined shapes like ellipses or circles, or freely drawing scribes (as they have prove to be largely used in interactive segmentation applications [47], [62]. The initialization requires the selection of at least one *object*, and, optionally, of one or more *background* areas.

**Figure 2:**
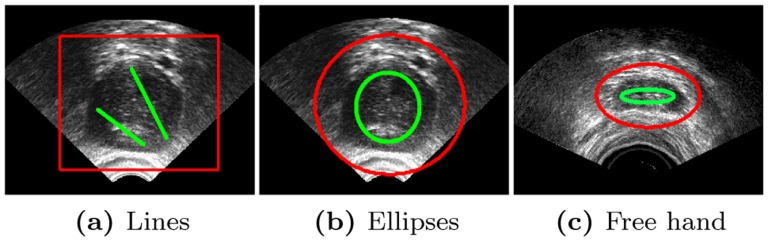
Different types of initialization using basic shapes like lines or ellipses, and free hand initialization. Yellow and light blue correspond to foreground and background labels respectively.

**Figure 3:**
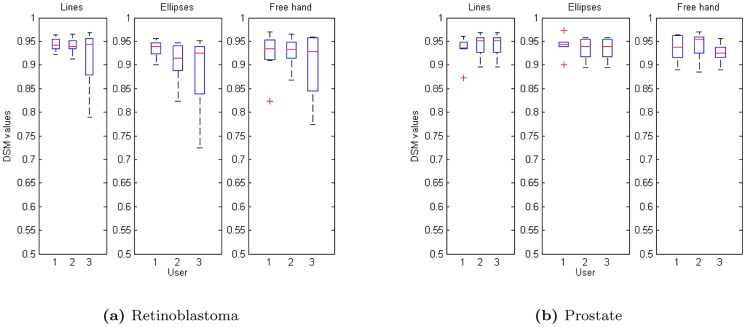
Box-plot of inter-user initialization variability and of the robustness with respect to different labels: central red mark is the median, edges of the box are the 25th and 75th percentiles, whiskers extend to extreme values, and outliers are plotted by a red cross.

We test the robustness of our method for both eye and prostate imaging. Some examples of different types of initial labels (lines, ellipses and free hand) are shown in Figure 2. Moreover, three different users have drawn the initial labels to study also the inter-user variability. Results are shown in the boxplots of Figure 3. For a given user, DCM is in average 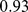 for both eye and prostate imaging with 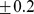 and 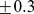 of variance respectively. Therefore, this demonstrate a high robustness as regards different types of initializations. Moreover, inter-user variability for a given label is also highly robust (around 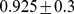). We applied the Wilcoxon two-sided rank sum test, a non-parametric alternative to paired-student test since it does not make any assumptions regarding the distributions of the data population, on DCM statistics. All tests accepted the null hypothesis at 5% significance level, that is, differences between each boxplot are not statistically significant. Therefore we can conclude that all types of initialization are valid for segmentation and that results are repeatable by different users.

Additional study concerning the spatial filling of the initial labels can be found in File S1 attached to the manuscript.

### Validation

**Figure 4:**
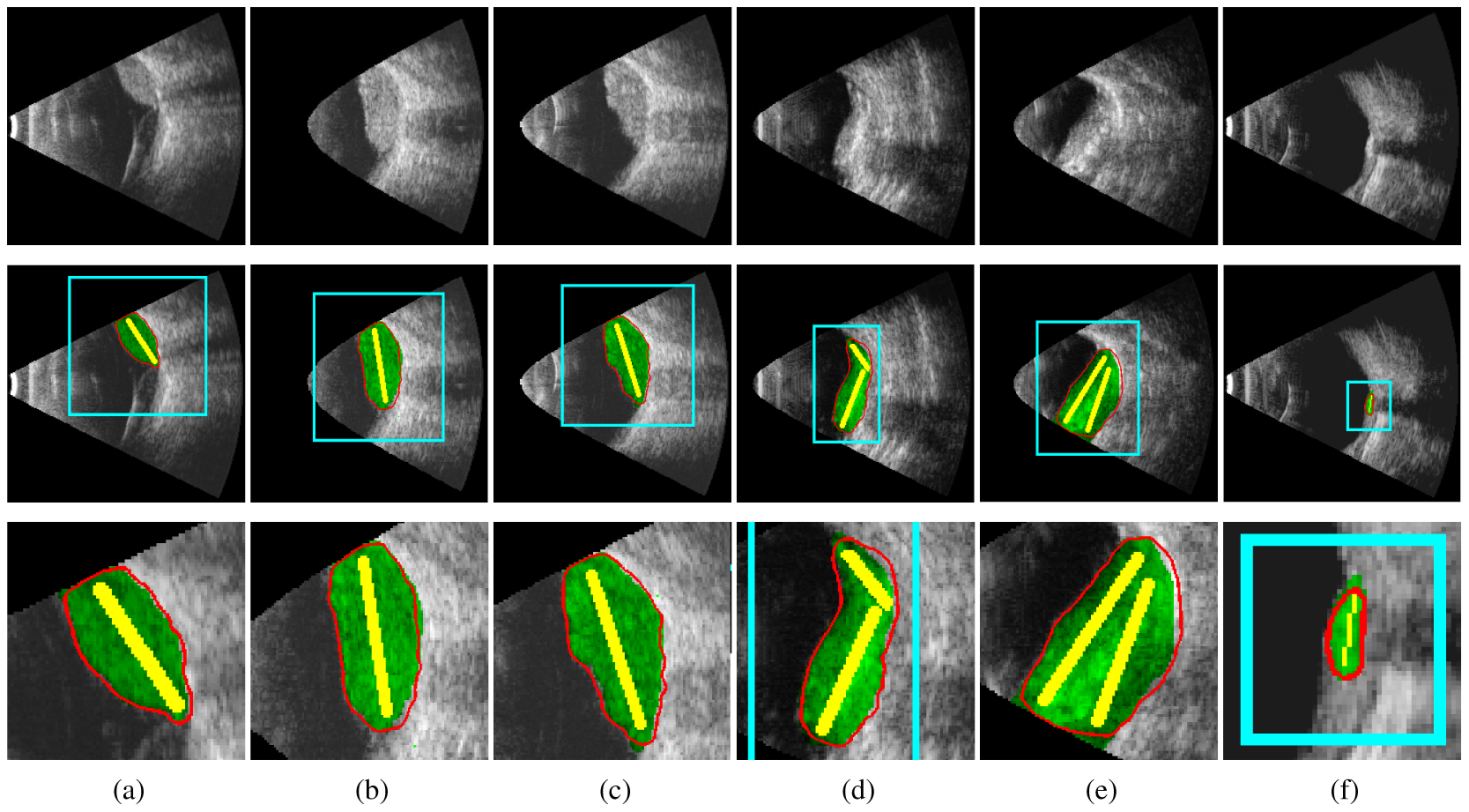
Eye tumor segmentation: ground truth is in transparent green, *fP-CMC* segmentation is in red, foreground and background labels are in yellow and light blue respectively.

**Figure 5:**
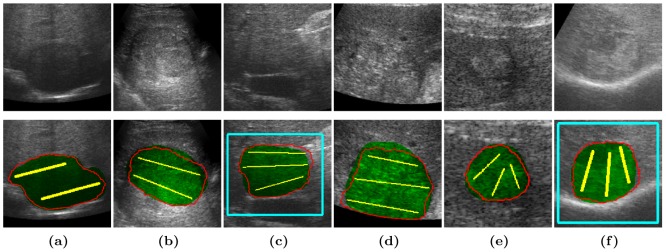
Liver tumor segmentation. Image resolution is 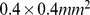 for (a), (b) and (c), and 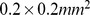 for (d), (e) and (f). Ground truth is in transparent green, *fP-CMC* segmentation is in red, foreground and background labels are in yellow and light blue respectively.

Quantitative assessment is presented for the eye, the liver, the prostate, and the fetus. The color code used in figures 4, 5, 7 and 9 is as follows: foreground and background labels are in yellow and light blue respectively, fP-CMC segmentation is in solid red contour, and ground truth is in transparent green (except for figure 7 where ground truth segmentation is not available, only the fetal head circumference value).

#### Segmentation of eye tumors

Segmentation results of US image segmentation are shown in Figure 4 (input images in the first line, segmentation results in the second one and zoom on the results in the third one). The Dice coefficient metric and computational time, in comparison with the previous *P-CMC* method [20], are presented in Table 3. DCM is 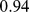 with a standard deviation of 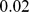 was obtained in average for the 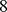 images of the eye data set. The speed-up of the novel method is clear visible from the reduced time of convergence and number of iterations presented. We measure the intra- and inter-observer variability of two raters that manually delineated the ground truth retinoblastoma. The inter-observer Dice average and variance is of 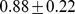 and the intra-observer variability is of 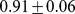. The *fP-CMC* Dice average of 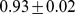 is good as compared to the variability measurements.

**Table 3:** Quantitatively evaluation of retinoblastoma segmentation against expert delineations used as ground truth.

Figure		4(a)	4(b)	4(c)	4(d)	4(e)	4(f)
**DCM**	*P-CMC*	0.95	0.93	0.94	0.89	0.95	0.72
	***fP-CMC***	0.96	0.95	0.95	0.93	0.95	0.90
**Iterations**	*P-CMC*	6217	4529	3031	3914	4287	1241
	***fP-CMC***	46	50	52	32	33	29
**Convergence Time (s)**	*P-CMC*	75	55	39	42	130	14
	***fP-CMC***	18	21	21	13	13	12

#### Segmentation of liver tumors

**Figure 6:**
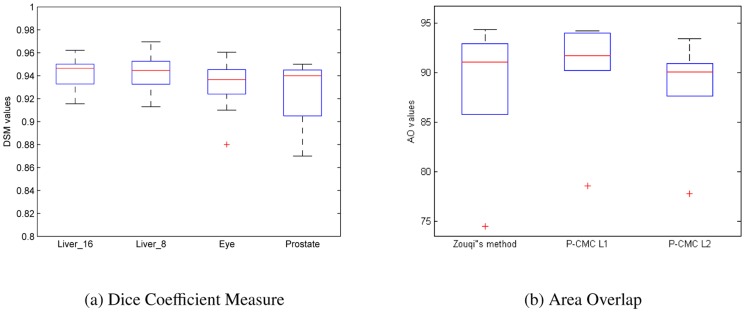
Box-plots of B-mode segmentations: (a) DCM values for liver tumors with a depth of 8 (Liver_8) and 16 (Liver_16), eye tumors and prostate; (b) Area overlap (AO) values in prostate segmentation of *fP-CMC* (using two different types of initial labels: *init 1* and *init2*) vs. GC-FIS [12].

A region of interest of 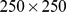 was defined from the original images (first row of Figure 5). Segmentation results are shown in second row of Figure 5 for different resolutions: (a) to (c) have a resolution of 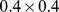 mm^2^ and (d) to (f) have a resolution of 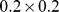 mm^2^. Similar results were obtained for all images in the US liver data set. Overlap measure and computational time are presented in Table 4. The average DCM of the 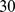 images of the liver data set is 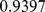 with standard deviation of 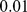. This performance proofs a good accuracy and high robustness for these challenging imaging. Moreover, as regards our previous method, the number of iterations till convergence and the computational time are reduced significantly, by a factor of 1000 and 15 respectively. A box-plot representation of the DCM is visualized in Figure 6, where the first box corresponds to the US liver data set of 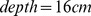 and the second one to the data set of 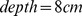. The same accuracy is almost reached for both image resolutions but slightly higher variability is obtained for acquisitions at 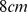 depth.

**Table 4:** Quantitatively evaluation of the liver tumors against manual segmentation by an expert radiologist as ground truth.

Figure		5(a)	5(b)	5(c)	5(d)	5(e)	5(f)
**DCM**	*P-CMC*	0.94	0.95	0.94	0.93	0.91	0.92
	***fP-CMC***	0.96	0.95	0.95	0.95	0.93	0.95
**Iterations**	*P-CMC*	17566	11786	10103	19163	17732	19605
	***fP-CMC***	48	85	80	48	43	84
**Convergence Time (s)**	*P-CMC*	300	245	359	379	471	281
	***fP-CMC***	20	35	33	20	18	35

#### Fetal head segmentation

**Figure 7:**
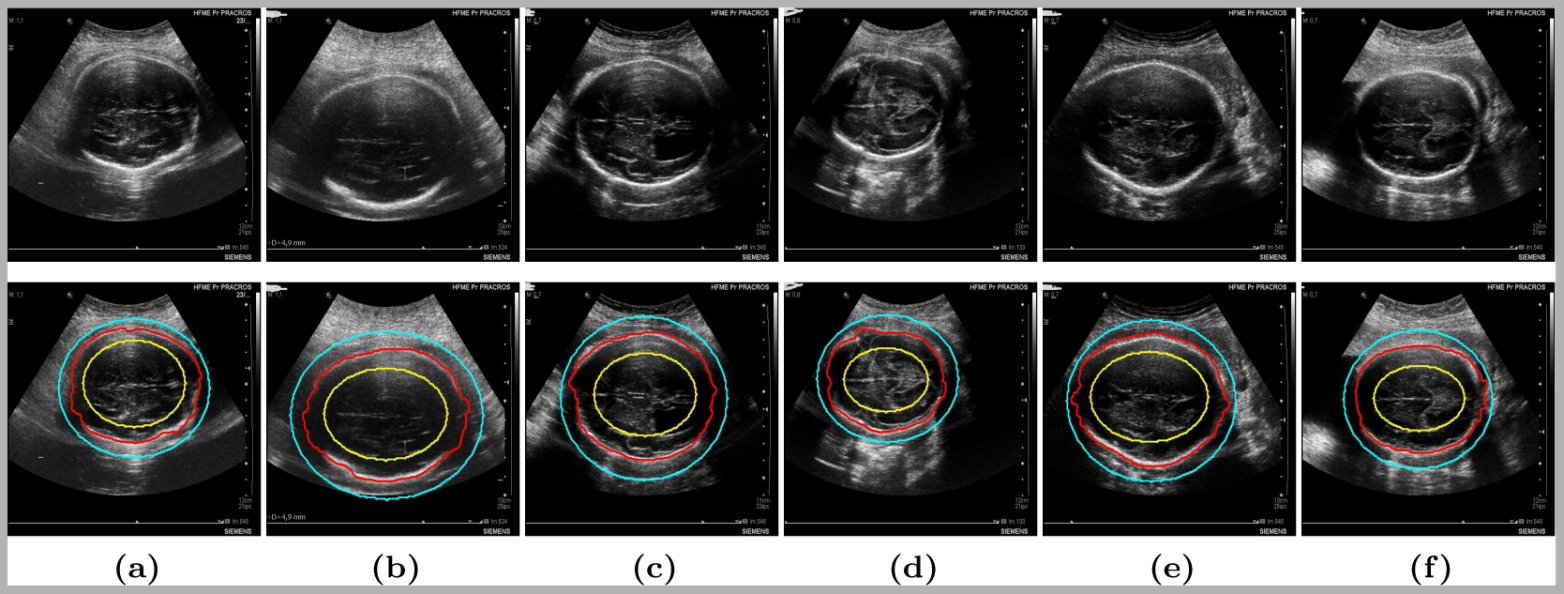
Fetal head segmentation: *fP-CMC* segmentation is in red, initial labels (ellipses) are in yellow and light blue for object and background, respectively.

We proceed to define two elliptic labels inside and outside the fetus head (see yellow and cyan contours in Fig. 7) by a mouse click (press, drag and release). Then, the head is segmented with the *fP-CMC* (red contour in Fig. 7). From there, the axis of elongation of the obtained binary segmentation is computed with the following formulation [63]:
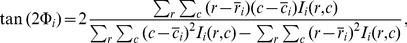
(19)where r, c corresponds to the rows and columns in the binary image I (1- for the object and 0 for the background) and 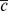, 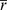 are the coordinates for the centers of mass. The axis of elongation will correspond to Occipito-Frontal Diameter (OFD) measure, and then Biparietal Diameter (BPD) is computed for the angle (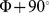), for the same center of mass. Finally, HC is computed from the BPD and OFD measurements using the expression [64]:
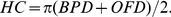
(20)


**Figure 8:**
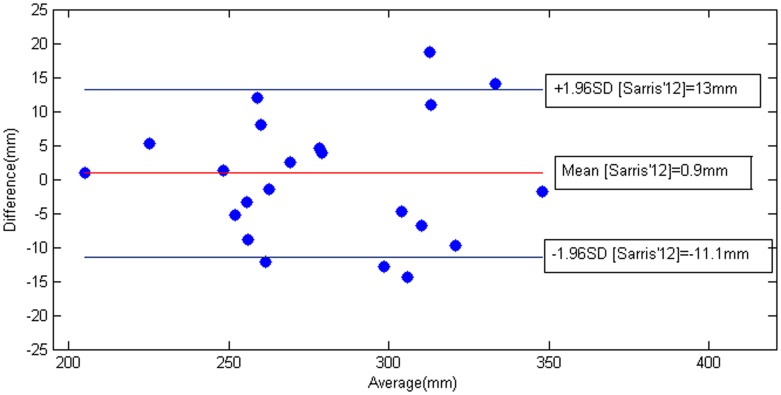
Inter-subject variability. Error measurements of our method w.r.t. the confidence interval at 95% and Bland-Altman plot of inter-subject variability from [65].

Some visual segmentation results are shown in Figure 7 with their corresponding quantitative measures as in Table 5. Estimated HC metric as compared with radiologist computation for all images is plotted in Figure 8 in form of Bland-Altman plot [64] where the error in the HC estimation (difference in mm) is represented as a function of the estimated HC measure (in mm). Further, we compare our estimation errors with the inter-user variability of HC measurement reported in previous studies [65]. Sarris et al [65] recently presented, among other fetal ultrasound measurements, a variability study of HC measurements (in mm) between 3 different users over 175 images from 140 fetuses (ranging from 14 to 41 weeks of gestational age). They reported an average variability of 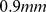 and the 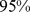 confidence intervals at 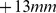 and 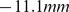. Our average error is smaller (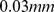) and our error distribution fall within those confidence interval. In this sense, we can say that our estimated HC is equivalent to the one measured by hand by the radiologist.

**Table 5:** Quantitative evaluation of the estimated fetal head circumference versus biometry provided by radiologists.

Figure		7(a)	7(b)	7(c)	7(d)	7(e)	7(f)
**HC (mm)**	*fP-CMC*	253	265	257	250	255	251
	***GT***	260	256	267	262	257	254
**Iterations**	***fP-CMC***	2	2	3	2	3	2
**Convergence Time (s)**	***fP-CMC***	7	6	8	6	8	8

#### Segmentation of prostate

In this application we compare our *fP-CMC* segmentation to previous published works [12] that are based on graph cut segmentation with a fuzzy interference system (*GC-FIS*). Both *fP-CMC* and *GC-FIS* methodologies are comparable in terms of interactivity and of semi-supervision with labels within a graph-cut based segmentation framework. However, contrary to our philosophy of considering speckle as image information, GC-FIS assumes a preprocessing step for image de-noising in order to reduce the speckle noise by using ‘stick filter’ and smooths the image. The user interaction in GC-FIS differs a lot of ours indeed. In GC-FIS, few points lying on the object contour must be selected first. These points are further used to generate the contours for the inside and the outside labels, being automatically defined in the normal direction and at a certain distance from the object contour. Segmentation is performed next, using the interactive graph-cut approach proposed by Boykov and Jolly in [21]. Please note that GC-FIS is thus based on a discrete optimization approach, while we have a continuous formulation. Finally, the contour is refined in a post processing step using a FIS, where each point is evaluated and marked as weak or strong boundary points. The misclassified (weak) points are used next to add more appropriate hard constraints and to further re-perform the segmentation.

**Figure 9:**
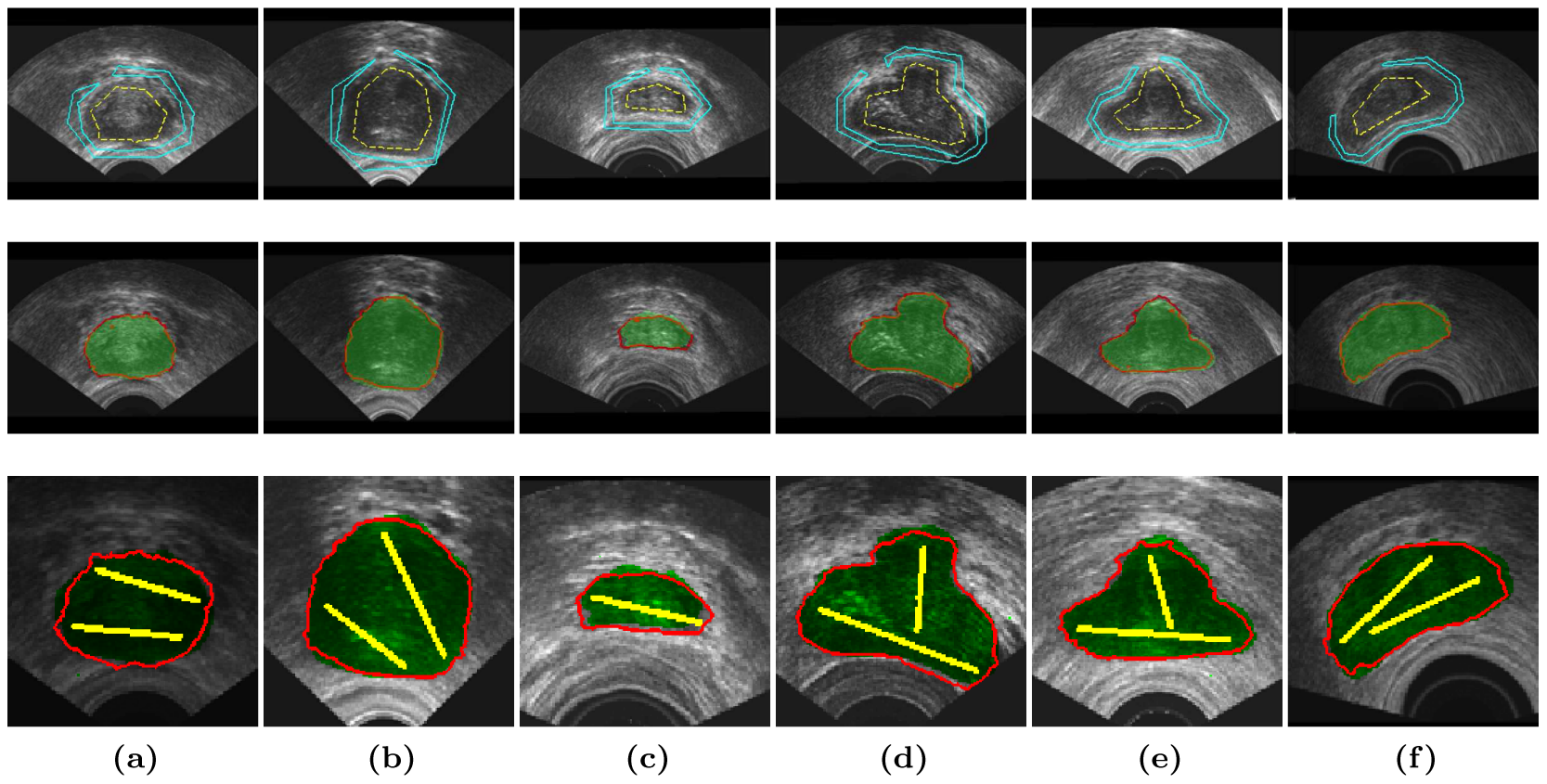
Segmentation of US prostate data set. 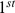
 and 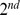 rows: initialization labels as in [12] (yellow for object and blue for background) and *fP-CMC-L1* segmentation in red. 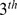 row: initial labels in yellow and *fP-CMC-L2* segmentation in red contour. Ground truth is in transparent green area. We refer to [12] for visual comparison with GC-FIS results.

For sake of comparison we force us to define labels as close as possible as defined by *GC-FIS*. Accordingly, in Fig. 9, for the initial labels shown in the 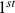 row we obtain the segmentation results as shown in the 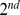 row. We will denote these results by *fP-CMC-L1*. Rather than using these *complex* labels, we will run our algorithm with much simple and intuitive initialization (as performed in all previous clinical applications), by straight lines for the foreground and without background labels. Results are denoted by *fP-CMC-L2* and shown in 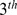 row of Fig. 9.

In Table 6 we present the quantitative results for both methods in terms of area overlap (AO). Results with *fP-CMC-L1* are very close to *GC-FIS* or slightly better: an overall accuracy average of 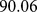 of *fP-CMC-L1* against 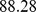 obtained with *GC-FIS*. We have considerably reduced the variability of the results, proving again the robustness of the *fP-CMC* (see boxplots in Fig. 6). Results with *fP-CMC-L2*, (average accuracy of 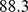) are slightly worse than *fP-CMC-L1* and equivalent to *GC-FIS*. We believe though that this decrease in accuracy is acceptable when considering the much more simple initialization step without background labels. The DCM boxplot for *fP-CMC-L2* are shown in Fig. 6.

**Table 6:** Comparison with GC-FIS [12] using overall accuracy (AO).

Figure	9(a)	9(b)	9(c)	9(d)	9(e)	9(f)
**AO-** ***fP-CMC-L1***	94.18	92.28	78.56	94.00	91.12	90.22
**AO-** ***fP-CMC-L2***	87.65	93.43	77.79	90.92	90.02	90.15
**AO [12]**	94.35	92.92	74.54	91.40	85.81	90.71

## Conclusion

The most important methodological contributions of our work are the use of a graph of intensity patches for representing the ultrasound image and a fast minimization scheme for the continuous min cut problem. This new formulation has considerably reduced the computation time as regards our previous version [20]. Intuitively, our proposed graph cut method can be seen as a heat diffusion process on the graph of image patches. The heat diffusion propagates the information of the inside and outside labels selected by the user. Regions of interest that wish to be segmented entirely depend on the choice of the labels, i.e. which image patches are selected to be diffused. In other words, our segmentation method can segment both homogeneous and heterogeneous US regions, offering high-quality results with a large flexibility to work on different targets.

In practice, most parameters are easily set. Actually, we found that for many different B-mode sequence and scanners, the same parameter values provided high accurate results (all parameters are equally set for all clinical applications presented). The only parameter that may required an initial fine tuning according to B-mode sequence is the scaling parameter 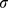 which is more sensitive when the contrast between foreground and background is low, but which can be overcame with a proper label initialization. We showed that despite the semi-supervision, our segmentation framework provides highly accurate and quantitatively similar results for different types of labels and users. Indeed, setting the initial labels (by few clicks, lines, circles, etc.) is extremely easy and fast. The user interaction in the label definition might be seen as a limitation of our method but, in our opinion, ultrasound image analysis is particularly suited for an interactive segmentation framework since, in clinical practice, physicians are used to extract simple biometric measures from few click interactions. Moreover, in cases where ultrasound image quality is really challenging, our framework allows the expert to define labels close to the final object thus providing not only a faster convergence but also more reproducible segmentations. Nevertheless, in an application basis, the label definition process can easily be automatized if needed [20].

We have evaluated the segmentation results of the *fP-CMC* framework in several clinical contexts (some of them aimed at quantitative validation purposes more than real clinical applications). We have reported segmentation accuracy in average of 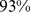 for all clinical applications (Fig. 6). This proofs a high practical value of our method in terms of flexibility and easy of use as compared to state of the art methods (limited and optimized for a specific organ and/or image sequence). Our interactive patch-based philosophy is rather different from most existing segmentation techniques for B-mode ultrasound imaging. Nevertheless, we have compared our method to the closest methodology in terms of interactive discrete graph cut segmentation [12] applied to the prostate.

We have presented a segmentation method for 2D US images since 2D US is widely accepted in clinical practice (except for the eye, all our images where in 2D). However, following the increasing number of 3D US imaging, the extension to 3D images is does not change the mathematics neither the algorithm introduced in this work. Plus, initialization could be easily extended to 3D, for instance considering a few group of 2D slices where the user clicks or to make use of 3D objects (sphere or ellipsoids) as initial labels. We assume that in 3D we would keep the same robustness as in the 2D case. However, this would require confirmation with new experiments, which is out of the scope of the paper.

Finally, we promote here the use of soft priors (semi-supervision with labels) for the ultrasound segmentation. Nevertheless, we agree that the use of strong shape, temporal and/or intensity, priors can be for a specific target more powerful that our soft priors. Note that strong priors can be easily included in our mathematical formulation. Future work goes into exploring other image features to be included in the patch representation (and the corresponding patch distance function) in order to better represent the echogenicity present in US imaging.

## Supporting Information

File S1 Extra validation of the initial label.
(PDF)
