# Supplementary material for: Semi-Supervised Segmentation of Ultrasound Images Based on Patch Representation and Continuous Min Cut
Source: PLoS One. 2014 Jul 10;9(7):e100972. doi: 10.1371/journal.pone.0100972 (PMC4091944; doi:10.1371/journal.pone.0100972)
Supplement: File S1 — Extra validation of the initial label. (PDF) [file pone.0100972.s001.pdf]

Supplementary material

In this section we evaluate how critical is the initialization in the sense of ”spatial filling” provided by the user delineation. We have defined ten initial labels, differing in volume, shape, position and number of labels. Thus the spatial filling of the labels is rather different. Segmentation results are shown in Table S1 and Table S2 for the segmentation of a simulated image (the ideal case when the object is homogeneous) and of an eye tumor (real case). In the first row of each table, an image where initial labels (yellow corresponds to the contour of the object label and blue - the contour of the background label) and the final segmentation results (in red) are superimposed; in the second row, the percentage volume of the label w.r.t. the target object; third row, the number of iterations till convergence; and fourth row, the Dice coefficient metric (DCM). In these application, the final contour and accuracy obtained are indeed very similar among a large variety of space occupying initial labels.

However, in some challenging cases, it will be necessary to ensure a minimal space filling in order to obtain good segmentation results. We test the label initialization on such a case, namely on a liver US image (Figure S1(a)), where by ”challenging case” we refer to images containing non-homogeneous object, and eventually corrupted by artifacts (such as shadows, speckle etc.). We start our study by initializing our algorithm with one line object and then we add lines one by one while recording the DCM values of the segmentation results against the ground truth. These information: the initial labels, segmentation results and the corresponding DCM values are shown in Figure S1. It can be noticed that even for such a challenging case, an initialization with only two line labels is enough to obtain a good DCM of 0.95. This value can be however improved (to 0.98 in this case) if some pixels in the corrupted regions (by shadow - Fig. S1(d) and by calcification - Fig. S1(f)) are marked as object pixels. This little improvement would come though at a cost of more trials. This example shows indeed the advantage of being semi-supervised, since the user will quickly add one more line and obtain good results already in a second trial. This easy semi-supervision allows us to obtain very good results in a wide range of applications and, to our knowledge, there are not other semi-automated or automated segmentation methods able to do so.

	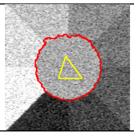	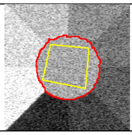	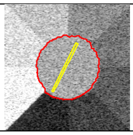	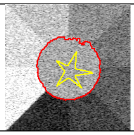	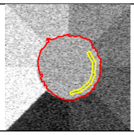	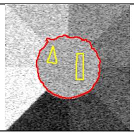	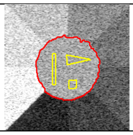	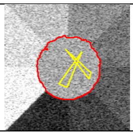	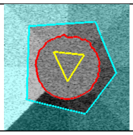	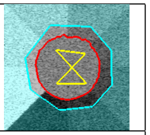
**Volume %**	8	48	4	11	5	14	8	8	13	14
**Iterations**	5	2	10	5	6	5	10	4	3	5
**DCM**	0.97	0.98	0.97	0.97	0.97	0.96	0.97	0.97	0.96	0.95

Table S1: Segmentation result variation w.r.t. label initialization on a simulated US image (initial labels are in yellow (object)/ blue (background) contours and the segmentation results in red contour).

	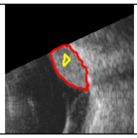	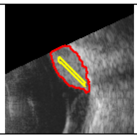	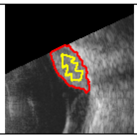	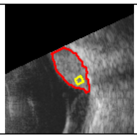	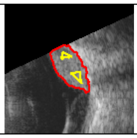	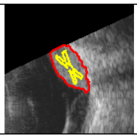	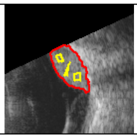	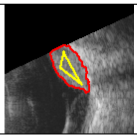	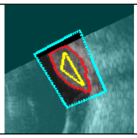	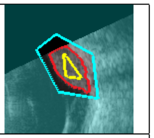
**Volume %**	5	12	27	4	9	17	11	19	17	15
**Iterations**	5	4	2	5	5	3	4	3	3	3
**DCM**	0.94	0.96	0.96	0.93	0.96	0.96	0.96	0.96	0.96	0.96

Table S2: Segmentation result variation w.r.t. label initialization on a real US image (initial labels are in yellow (object)/ blue (background) contours and the segmentation results in red contour).

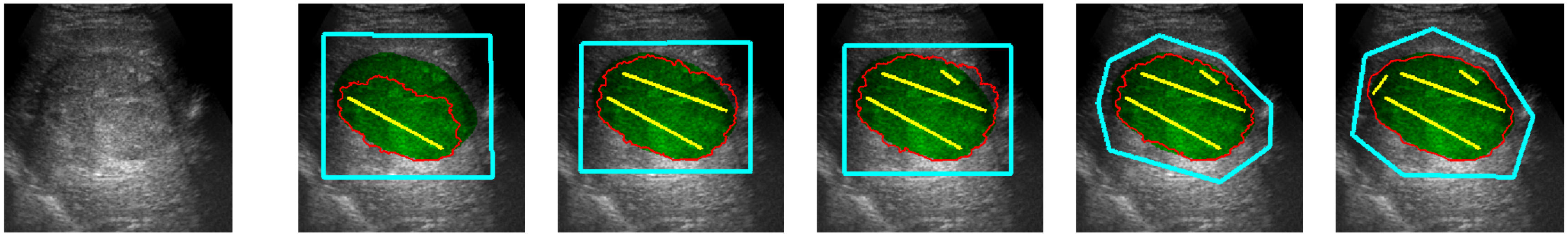

(a) Original US liver image (b) DCM = 0.76 (c) DCM = 0.95 (d) DCM = 0.95 (e) DCM = 0.96 (f) DCM = 0.98

Figure S1: Initial label's study for in liver US image.
